# Combined Application of Bevacizumab and Mitomycin C or Bevacizumab and 5-Fluorouracil in Experimental Glaucoma Filtration Surgery

**DOI:** 10.1155/2018/8965709

**Published:** 2018-09-09

**Authors:** Lei Zuo, Jianhong Zhang, Xun Xu

**Affiliations:** ^1^Department of Ophthalmology, Shanghai Fourth People's Hospital, Shanghai 200081, China; ^2^Department of Ophthalmology, Shanghai General Hospital, Shanghai Jiao Tong University School of Medicine, Shanghai 200080, China

## Abstract

The present study aimed at observing the effect of a single subconjunctival injection of bevacizumab (BVZ) combined with 5-fluorouracil (5-Fu) or mitomycin C (MMC) on the antiscarring effect of glaucoma filtration surgery (GFS). The inhibitory effect of combined BVZ and 5-Fu in retinal pigment epithelial cells on vascular endothelial growth factor (VEGF) levels was demonstrated through *in vitro* experiments. Combined BVZ and 5-Fu and combined BVZ and MMC inhibited cell cycle, induced apoptosis, and inhibited human umbilical vein endothelial cell migration. Also, the cytotoxicity of combined BVZ and 5-Fu was lower. In animal experiments, the observation of filtering bleb survival, hematoxylin and eosin and Masson staining of filtering bleb scars, and mRNA expression levels of fibrosis markers in filtering blebs showed that combined BVZ and 5-Fu had a better antiscarring effect compared with single drugs; however, the antiscarring effect of combined BVZ and MMC was not significantly different from MMC. Therefore, the findings of this study provided more reference for the clinical use of adjuncts to inhibit scarring after GFS and helped understand the regulatory effect of combined anti-VEGF antibody BVZ and antimetabolites on wound healing more comprehensively.

## 1. Introduction

Glaucoma is a serious irreversible optic neuropathy that ultimately causes blindness. When maximum drug therapy cannot control intraocular pressure in patients with glaucoma, laser therapy or surgery must be performed. Glaucoma filtration surgery (GFS) is currently one of the most effective methods for treating glaucoma [1, 2]. Unlike with most operations, the success of GFS is achieved by inhibiting wound healing [3]. In the 1990s, antimetabolic drugs, such as fluorouracil (5-Fu) and mitomycin (MMC), were used to reduce scar formation after a trabeculectomy and to maintain continuous unobstructed filtering, thereby improving the success rate of surgery [4]. However, despite their effectiveness, these drugs were related to several types of life-threatening visual acuity complications [5, 6].

Vascular endothelial growth factor (VEGF) is a signaling protein that promotes vascular endothelial growth and permeability [7]. Also, it has a key role in angiogenesis and embryonic angiogenesis. Further, it participates in pathological angiogenesis, such as tumor growth [8, 9] and ocular diseases. VEGF concentration increases in all ocular diseases involving neovascularization and/or inflammation, such as proliferative diabetic retinopathy [10], neovascular glaucoma [11], uveitis [12], and age-related macular degeneration [13]. In addition, VEGF is related to fibrosis and inflammation [14, 15].

Bevacizumab (BVZ) is a synthetic anti-VEGF monoclonal antibody [16, 17]. Recent studies found that VEGF concentration increased and VEGF levels in the aqueous humor were upregulated in patients after glaucoma surgery. This postsurgery upregulation could be suppressed by the anti-VEGF antibody, BVZ, administered during the surgery [18]. BVZ reduces the migration of VEGF into injured vessels and significantly inhibits scar formation during wound healing [18, 19]. Furthermore, the watertight suture in the conjunctiva can antagonize BVZ-induced delayed healing of the conjunctival wound [20]. Subconjunctival injection of BVZ could reach an effective level in the intraocular tissues in the treated eyes [21]. On the basis of these findings, doses of BVZ were injected to inhibit scar formation after trabeculectomy.

Kahook et al. first reported and used the anti-VEGF antibody as a potential wound-healing regulator; 1 mg BVZ was injected around a filtering bleb using a fine needle after MMC application failed [22]. In other cases, Grewal reported that a subconjunctival injection of BVZ helped save the failed filtering blebs [23]. Some animal studies also demonstrated the effectiveness of BVZ combined with 5-Fu (BVZ + 5-Fu) in preventing scarring after GFS [24]. Some animal studies used a sustained-release device carrying MMC in trabeculectomy, suggesting that BVZ combined with MMC (BVZ + MMC) had synergistic effects [25]. Although reports on the antiproliferative effect and toxicity of BVZ on ocular cells have been reported [26], the role of BVZ + 5-Fu and BVZ + MMC in human ocular cells has rarely been investigated, and the comparison of the application of BVZ + 5-Fu or BVZ + MMC in experimental GFS surgery and their effectiveness remains unclear.

This study evaluated the safety and effect of a single subconjunctival injection of BVZ combined with 5-Fu or MMC on the antiscarring effect of GFS and compared them with the use of uncombined agents in *in vitro* and animal experiments. Cytotoxicity, the survival time of filtering blebs, and mRNA expression levels of fibrosis markers were observed. These findings provided a reference for glaucoma treatment in clinical surgeries.

## 2. Materials and Methods

### 2.1. *In Vitro* Experiment

#### 2.1.1. Drugs and Reagents

Medium, antibiotics, trypsin (1 : 250), recombinant human VEGF, 3-(4,5-dimethylthiazol-2-yl)-2,5-diphenyltetrazolium bromide (MTT), and heat-inactivated fetal bovine serum (FBS) were purchased from Invitrogen (CA, USA). Endothelial cell culture medium was purchased from PromoCell GmbH (Heidelberg, Germany). Bevacizumab (Avastin), PhosSTOP, and protease inhibitors were purchased from Roche (Basel, Switzerland). The enzyme-linked immunosorbent assay (ELISA) kit was purchased from R&D Systems (MN, USA). The Bradford protein assay was purchased from Bio-Rad (Hercules, CA, USA). Fluorouracil (25 mg/mL) was purchased from Shanghai Xudong Haipu Pharmaceutical Co. Ltd. (China), and mitomycin was purchased from Zhejiang Hisun Pharmaceutical Co., Ltd. (China). Phosphate-buffered saline (PBS) and 0.9% sodium chloride were purchased from Baxter Healthcare Ltd. Human retinal pigment epithelial cells (ARPE-19) and human umbilical vein endothelial cells (HUVECs) were cultured in DMEM/F12 culture medium containing 10% FBS and antibiotics, respectively. ARPE-19 and HUVEC lines were purchased from American Type Culture Collection (VA, USA).

#### 2.1.2. Detection of Cytotoxicity/Proliferation

A single-cell suspension within the logarithmic growth period cultured under normal conditions was inoculated into a six-well culture plate at a cell density of 5 × 10^4^ cells per well. After synchronization using serum-free RPMI 1640 culture medium, ARPE-19 cells were incubated with 0.05, 0.5, or 5 mg/mL 5-Fu; 0.0002, 0.002, or 0.02 mg/mL MMC; 0.025, 0.25, or 2.5 mg/mL BVZ; 0.05, 0.5, or 5 mg/mL 5-Fu + 2.5 mg/mL BVZ; 0.0002, 0.002, or 0.02 mg/mL MMC + 2.5 mg/mL BVZ; or PBS as the control for 24 h, after which they were washed with PBS. Fresh serum-free medium with or without 0.5 mg/mL MTT was added to the cells. After incubating for 2 h, the colorimetric analysis was conducted to determine formazan extraction and ELISA (Emax, Molecular Devices Corporation, CA, USA) was conducted to measure the absorbance of each well at 570 nm [27].

#### 2.1.3. VEGF Level Determination

ARPE-19 cells were treated with 5 mg/mL 5-Fu, 0.02 mg/mL MMC, 2.5 mg/mL BVZ, 5 mg/mL 5-Fu + 2.5 mg/mL BVZ, 0.02 mg/mL MMC + 2.5 mg/mL BVZ, or PBS as the control. After 24 h, 200 *μ*L of the supernatant per well was collected and analyzed using a VEGF ELISA Kit (R&D Systems) according to the manufacturer's protocol [28].

#### 2.1.4. Assessment of Cell Cycle Changes Using Flow Cytometry with Propidium Iodide Staining

A single-cell suspension during the logarithmic growth period and cultured under normal conditions was inoculated into a six-well culture plate at a cell density of 5 × 10^4^ cells per well. After synchronization using serum-free RPMI 1640 culture medium, ARPE-19 cells were incubated with 5 mg/mL 5-Fu, 0.02 mg/mL MMC, 2.5 mg/mL BVZ, 5 mg/mL 5-Fu + 2.5 mg/mL BVZ, 0.02 mg/mL MMC + 2.5 mg/mL BVZ, or PBS as the control for 48 h. Each application was done in triplicate. The cells were collected, washed with PBS, counted, adjusted into a single-cell suspension at 1 × 10^6^/mL, fixed with 70% ethanol, preserved at 4°C, and washed with PBS to remove the fixative before staining. RNase A (100 *μ*L) was added to the cell suspension. The cells were incubated in a 37°C water bath for 30 min, and 400 *μ*L of propidium iodide (PI) was added and mixed for staining. The suspension was then incubated again in the dark at 4°C for 30 min, and the cells were detected using flow cytometry. The percentage of cells in each cycle was fitted according to the cell distribution diagram of relative DNA content [29].

#### 2.1.5. Detection of Cell Apoptosis by Annexin V-FITC Staining

ARPE-19 cells were treated with 5 mg/mL 5-Fu, 0.02 mg/mL MMC, 2.5 mg/mL BVZ 5 mg/mL 5-Fu + 2.5 mg/mL BVZ, 0.02 mg/mL MMC + 2.5 mg/mL BVZ, or PBS as the control and incubated at 37°C in 50 mL/LCO_2_ for 48 h. Each application was done in triplicate. The cells were collected and washed with PBS. A binding buffer (500 *μ*L) was added to suspend the cells. Then, 5 *μ*L of Annexin V-FITC was added to the suspension and mixed, after which 5 *μ*L of PI was added and mixed. The cells were incubated in the dark at room temperature for 15 min, and the apoptotic cells were detected using flow cytometry [30].

#### 2.1.6. Migration Analysis of Endothelial Cells

HUVECs were cultured *in vitro* to 80% density. A 1 mm pipette tip was used to make three equidistant scratches perpendicular to the bottom of the cell culture dish. PBS was used to remove the cells floating on the scratches, and images using six different fields of vision were taken using the Leica DM IRB microscope (40×). The time point was set at 0 h and the cells were treated with 0.05 mg/mL 5-Fu, 0.0002 mg/mL MMC, 2.5 mg/mL BVZ, 0.05 mg/mL 5-Fu + 2.5 mg/mL BVZ, or 0.0002 mg/mL MMC + 2.5 mg/mL BVZ. The treatments were combined with 40 ng/mL VEGF; 40 ng/mL VEGF alone was added into 0.2% FBS culture medium as the control. The control group without drug intervention was set. Images using the Leica DM IRB microscope were taken after culturing for 24 h. Each treatment culture was duplicated, and the same area of each duplicate was selected for photographing. The scratch area at each time point was analyzed using the ImageJ software. The wound closure rate = (area of the wound at 0 h − area of the wound at 24 h)/area of the wound at 0 h [31].

### 2.7. Animal Experiment

#### 2.7.1. Animal Model

Sixty healthy male New Zealand white rabbits (aged 12–14 weeks and weighing 2.0–2.5 kg each) were used in this study. All animal experiments were approved by the Shanghai Science and Technology Committee (permit number: SYXK[hu]2015-0014) and were in accordance with the Association for Research in Vision and Ophthalmology declaration of animal use. The experimental animals were kept under a 12/12 h light/dark cycle with random feeding. They were domesticated for 1 week before the experiment.

Trabeculectomy surgery was performed according to the standard protocol. An intramuscular injection of 50 mg/kg ketamine hydrochloride (Gutian Pharmaceutical Company, Fujian, China) and 25 mg/kg chlorpromazine hydrochloride (Gutian Pharmaceutical Company) was administered for general anesthesia. Proparacaine hydrochloride eyedrops (Alcon USA, TX, USA) were applied as local anesthesia. The conjunctiva was separated along the corneal limbus, and then a scleral flap of 3 × 4 mm^2^ with the corneal limbus as the base and a thickness of one-half of the sclera was made. The iris root was excised after a trabecular meshwork of 1.5 × 2 mm^2^ was removed. The scleral flap was sutured with a 10-0 nylon thread, and the conjunctiva was sutured to a watertight seal [32]. The surgery was performed by an experienced physician.

All New Zealand white rabbits received GFS in their right eye and were randomly divided into the following six groups (*n*=10):

  Group 1. The control group: GFS, no ancillary drugs;
  Group 2. 5-Fu treatment group: at the end of GFS, 0.1 mL (50 mg/mL) of 5-Fu was injected under the conjunctiva next to the filtering bleb; attention was paid to avoid 5-Fu from entering the eye.
  Group 3. MMC group: before resection of the sclera and trabecular tissue, an absorbent cotton pad of 1 × 4 mm^2^ (0.2 mg/mL) was placed between the scleral bed and the scleral flap and covered with the conjunctiva and Tenon's capsule. Attention was paid to avoid the contact between the conjunctival flap and the cotton pad. After 3 min, the area was washed with 30 mL of balanced salt solution. The sclera was then resected, and the peripheral iridectomy was performed. The conjunctival incision was tightly sutured [33].
  Group 4. BVZ treatment group: at the end of GFS, 0.1 mL (25 mg/mL) of BVZ was injected under the conjunctiva next to the filtering bleb.
  Group 5. 5-Fu + BVZ treatment group: at the end of GFS, 0.1 mL of 5-Fu and 0.1 mL of BVZ were injected under the conjunctiva into one side of the filtering bleb.
  Group 6. MMC + BVZ treatment group: an MMC cotton pad (0.2 mg/mL) was placed for 3 min; after the surgery, 0.1 mL of BVZ was injected under the contralateral conjunctiva of the filtering blebs.

#### 2.7.2. Clinical Observation

A Kaplan–Meier survival curve (56 days of observation time) was drawn according to the survival time of the filtering blebs in each group [34]. The failure of a filtering bleb was defined as a flat, neovascularized, and scared filtering bleb with a deep anterior chamber [35]. The incidence of complications, such as wound leakage and corneal opacity, was observed.

#### 2.7.3. Polymerase Chain Reaction Analysis

Pentobarbital sodium (60–150 mg/kg) was injected intravenously 28 and 56 days after the surgery, and the filtering bleb tissue was removed. A polymerase chain reaction (PCR) analysis (*n*=3) was conducted to quantitatively analyze the expression of collagen I and fibronectin [24]. Total mRNA of the filtering bleb tissue was extracted and separated using TRIzol reagents (Invitrogen), and cDNA was synthesized using the Tetro cDNA Synthesis Kit (Bioline, London, UK). The mRNA expression level was detected by real-time (RT) PCR using the SensiFAST SYBR Hi-ROX Kit (Bioline) and analyzed with ABI Prism 7500 (SDS Software, USA).  Table 1 lists the primer sequences. The primers and probes for RT-PCR were designed by Shanghai Generay Biotech Co., Ltd. The expression levels of type I collagen and fibronectin mRNA were normalized using mRNA of glyceraldehyde-3-phosphate dehydrogenase (GAPDH).

#### 2.7.4. Tissue Sections

Pentobarbital sodium (60–150 mg/kg) was injected intravenously to remove the eyeball 56 days after the surgery. After denucleation, all eyeballs were fixed in the formalin acetate alcohol solution for 24 h, preserved in 70% ethanol, and fixed with paraffin. Sequential 5 *µ*m sections were prepared and stained with hematoxylin and eosin (H&E) for histological observation and Masson for detection of collagen deposition [36] (*n*=4).

#### 2.7.5. Statistical Analyses

The mean ± standard deviation was used to describe the variables. When the variance was homogeneous, the LSD and SNK tests of the analysis of variance were used. When the difference was inhomogeneous, the differences between experimental groups were analyzed using the rank-sum test. The Kaplan–Meier survival analysis and Mantel–Cox log-rank test were used to analyze the differences in survival time of the filtering blebs. All statistical analyses were carried out using SPSS 13.0 (SPSS Inc., IL, USA). A *P* < 0.05 was considered to be statistically significant.

## 3. Results

### 3.1. *In Vitro* Experiments

#### 3.1.1. Cytotoxicity Analyses

**Figure 1:**
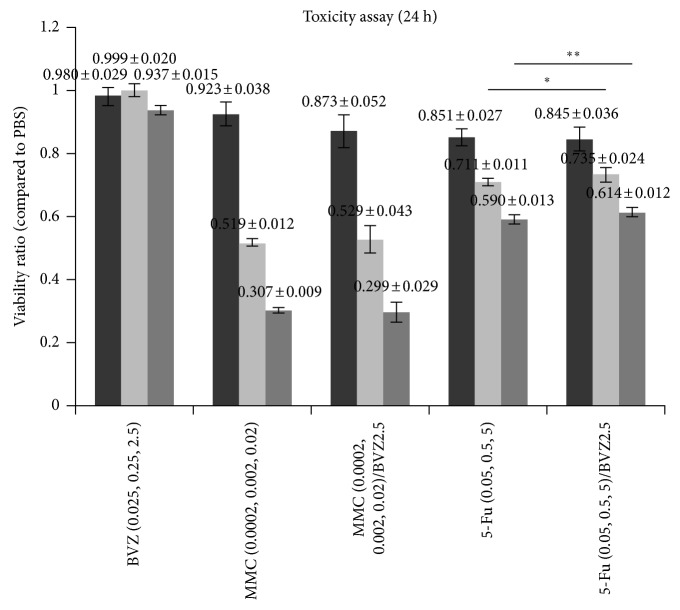
Cell viability of ARPE-19 after treatment with bevacizumab (BVZ), mitomycin C (MMC), 5-fluorouracil (5-Fu), MMC + BVZ, and 5-Fu + BVZ. The cell viability of the control group was set at 100%. Unit: mg/mL. ^*∗*^*P* < 0.05, ^*∗∗*^*P* < 0.01.

After adding 0.025, 0.25, and 2.5 mg/mL BVZ to the cultured ARPE-19 cells, no significant cytotoxicity was observed compared with that in the PBS group. After 0.0002, 0.002, and 0.02 mg/mL MMC were added, the viability of the ARPE-19 cells was significantly lower than that in the PBS group, with no significance difference between the MMC and MMC + BVZ groups. When 0.05, 0.5, and 5 mg/mL 5-Fu were added, the number of apoptotic ARPE-19 cells significantly increased. However, when 0.5 and 5 mg/mL 5-Fu + BVZ were added, the survival of ARPE-19 cells significantly increased (Figure 1).

#### 3.1.2. Detection of VEGF Level in Retinal Pigment Endothelium Cells

**Figure 2:**
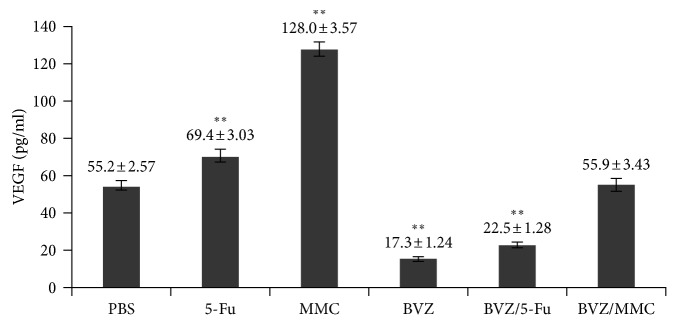
Effects of bevacizumab (BVZ), mitomycin C (MMC), 5-fluorouracil (5-Fu), MMC + BVZ, and 5-Fu + BVZ on vascular endothelial growth factor (VEGF) levels in retinal pigment endothelium (RPE) cells. ^*∗∗*^*P* < 0.001.

The ELISA method was used to analyze the VEGF levels in the medium to be able to detect VEGF expression in retinal pigment endothelium (RPE) cells after drug intervention. After culturing for 24 h, the VEGF levels in RPE cells in the BVZ and BVZ + 5-Fu groups were significantly lower than that in the control group, while the VEGF levels in the 5-Fu and MMC groups significantly increased (Figure 2).

#### 3.1.3. Cell Cycle and Apoptosis

**Figure 3:**
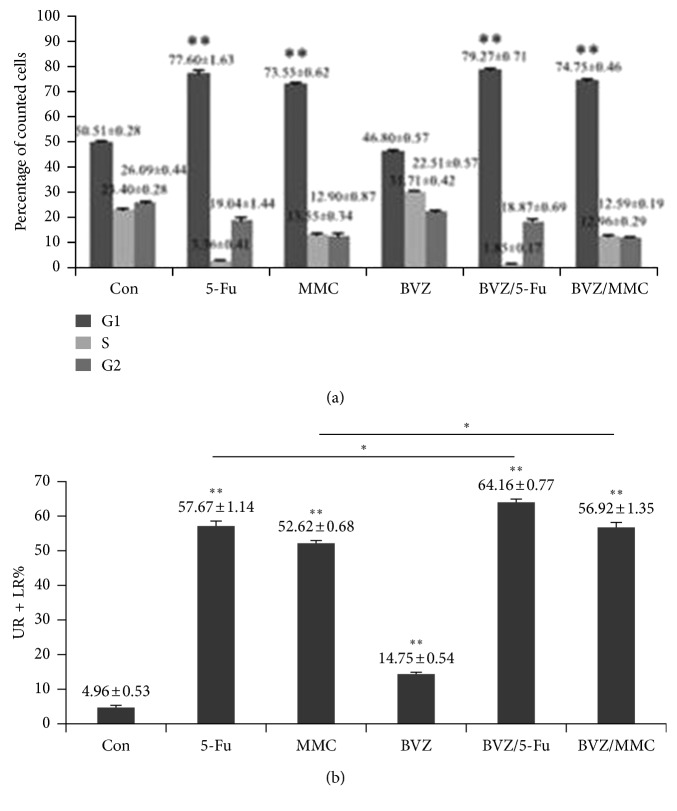
Analysis of the cell cycle and apoptosis of retinal pigment endothelium (RPE) cells. (a) Percentage of RPE cells in G1/G0, G2/M, and S phases after 48 h of incubation in PBS control, 5 mg/mL 5-fluorouracil (5-Fu), 0.02 mg/mL mitomycin C (MMC), 2.5 mg/mL bevacizumab (BVZ), 2.5 mg/mL BVZ + 5 mg/mL 5-Fu, and 2.5 mg/mL BVZ + 0.02 mg/mL MMC. ^*∗∗*^*P* < 0.001. (b) Effects of 48 h of incubation in the same concentrations of drugs on RPE cell apoptosis. ^*∗*^*P* < 0.01.

The effects of 5-Fu, MMC, BVZ, BVZ + 5-Fu, and BVZ + MMC on the cell cycle and apoptosis of RPE cells were evaluated using flow cytometry. The results showed that the proliferation of RPE cells was significantly inhibited after 5-Fu, MMC, BVZ + 5-Fu, or BVZ + MMC was added to RPE cells and incubated for 48 h compared to with those in the control group (Figure 3(a)). Treatment with 5-Fu, MMC, BVZ, BVZ + 5-Fu, or BVZ + MMC could significantly induce RPE cell apoptosis, and the inhibitory effect of the combined drugs was higher than that of the single drugs (Figure 3(b)).

#### 3.1.4. Cell Migration

**Figure 4:**
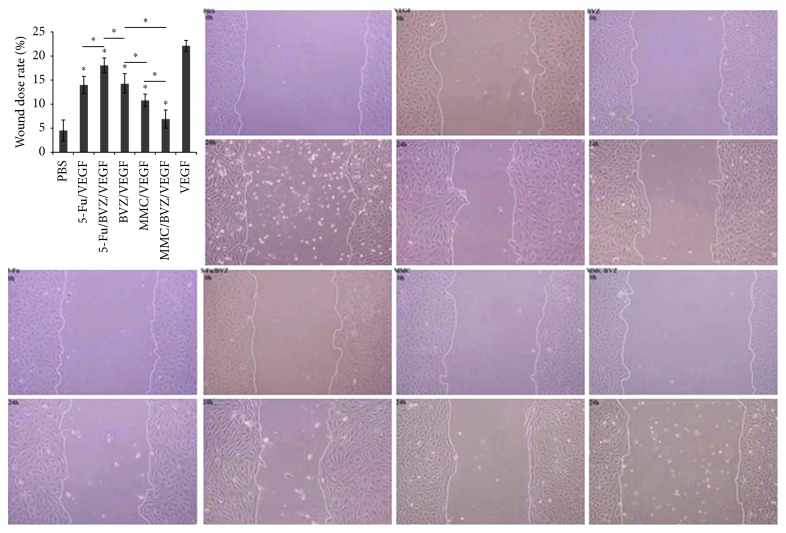
Cell migration analysis. The scratch-wound assay of human umbilical vein endothelial cells (HUVECs) after treatment with bevacizumab (BVZ), mitomycin C (MMC), 5-fluorouracil (5-Fu), MMC + BVZ, and 5-Fu + BVZ on the migration of endothelial cells under the action of vascular endothelial growth factor (VEGF). (*P* < 0.05).

The scratch-wound assay was used to evaluate the inhibitory effects of 5-Fu, MMC, BVZ, BVZ + 5-Fu, and BVZ + MMC on the migration of HUVECs.  Figure 4 shows the changes in the migration of HUVECs after 24 h under the action of VEGF and 5-Fu, MMC, BVZ, BVZ + 5-Fu, or BVZ + MMC. The wound closure rate of cells in the VEGF control group was 0.2215 ± 0.0117 after 24 h, and that of cells in the negative control group without drug intervention was 0.0454 ± 0.0216. The rate in the 5-Fu/VEGF, 5-Fu/BVZ/VEGF, BVZ/VEGF, MMC/VEGF, and MMC/BVZ/VEGF groups was 0.1402 ± 0.0183, 0.1811 ± 0.0147, 0.1438 ± 0.0194, 0.1086 ± 0.0132, and 0.0695 ± 0.0191, respectively. 5-Fu, MMC, BVZ, 5-Fu + BVZ, and MMC + BVZ could significantly inhibit the migration of HUVECs under the action of VEGF, and the inhibitory effect of MMC + BVZ was higher than that of MMC or BVZ alone (Figure 4).

### 3.2. Animal Experiments

#### 3.2.1. Survival of Filtering Blebs

**Figure 5:**
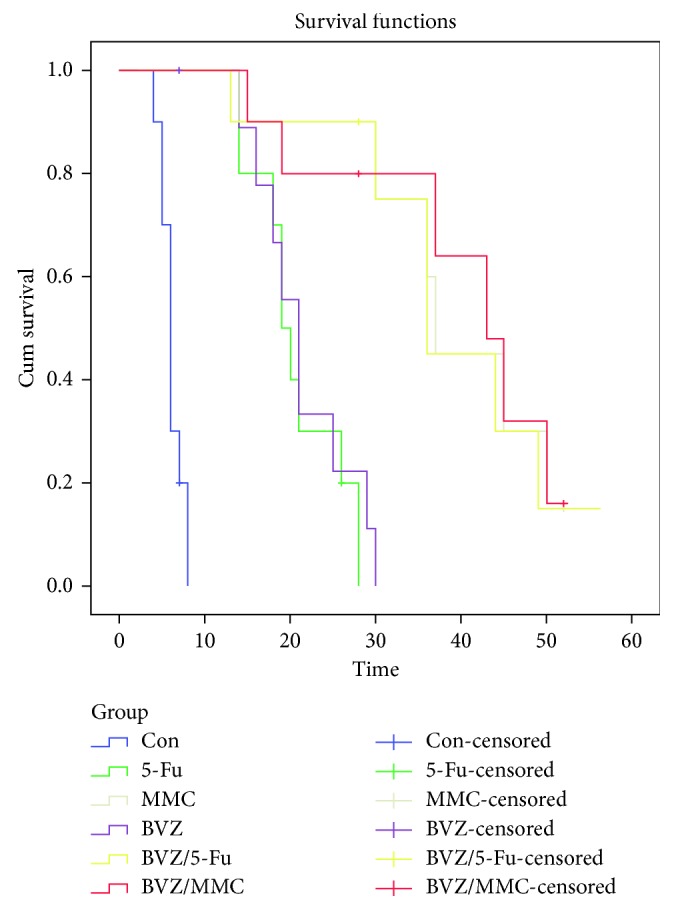
Kaplan–Meier analysis of the survival time of filtering blebs in each group.

The average survival time of filtering blebs in groups 1 (control), 2 (5-Fu), 3 (MMC), 4 (BVZ), 5 (BVZ + 5-Fu), and 6 (BVZ + MMC) was 6.3 ± 0.7, 22.4 ± 2.7, 35.5 ± 5.0, 23.8 ± 2.9, 36.0 ± 5.2, and 35.2 ± 5.6 days, respectively (Figure 5). The Kaplan–Meier analysis showed significant differences among the six groups (log-rank = 46.18; *P* < 0.001). The survival time of filtering blebs in the BVZ + 5-Fu group was significantly longer than that in the BVZ, control, and 5-Fu groups, with no significant difference compared with the MMC group. The survival time of filtering blebs was significantly longer in the BVZ + MMC group than in the control, 5-Fu, and BVZ groups, with no significant difference compared with that in the MMC or BVZ + 5-Fu group (Table 2).

#### 3.2.2. Bleb Vascularity

**Figure 6:**
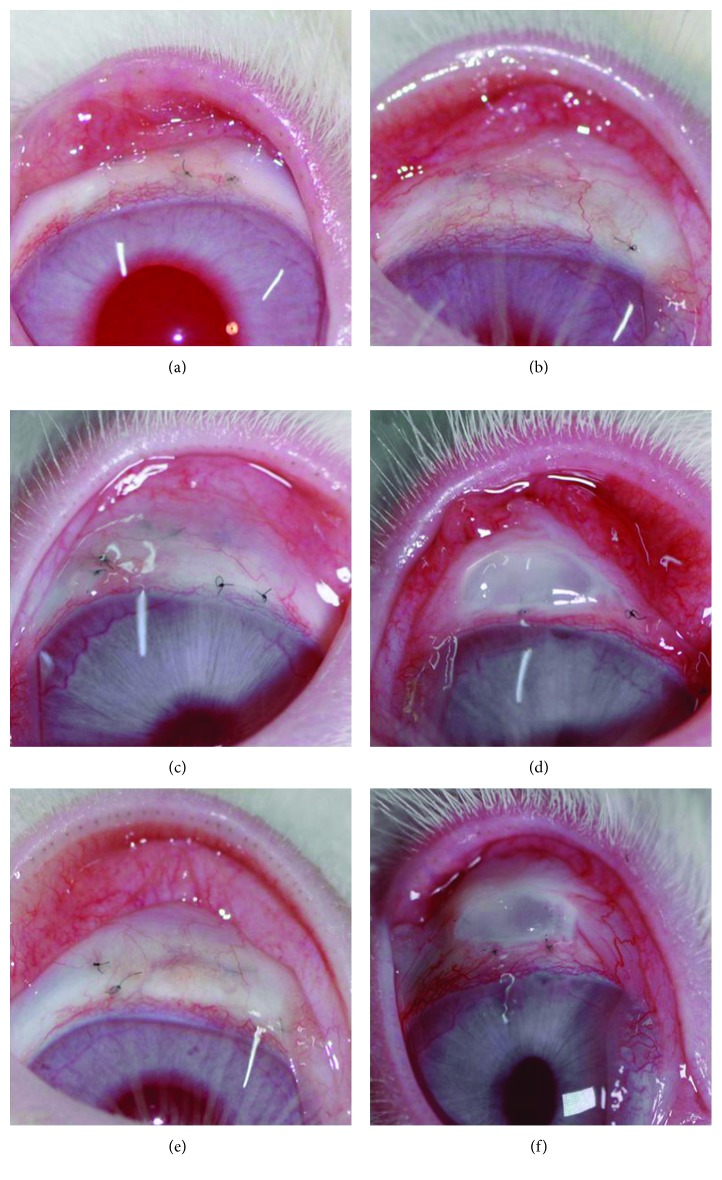
Bleb photograph examples in rabbit subconjunctival scar models 20 days after the surgery. Compared with the control group (a), 5-fluorouracil (5-Fu) treatment group (b) had a nearly normal distribution of blood vessels with filtering blebs low and flat. Compared with the control group (a), bevacizumab (BVZ) treatment (c) and 5-Fu + BVZ groups (e) showed slightly higher and diffused filtering blebs, and the distribution of blood vessels was not obvious. Compared with the control group (a), mitomycin C (MMC) (d) and MMC + BVZ groups (f) showed more conjunctival blood vessels with obviously bulged filtering blebs.

Figure 6 shows the bleb vascularity in the six groups of filtering blebs. No complications, such as wound leakage, encysted bleb, or corneal opacity, were observed during the study.

#### 3.2.3. Histopathological Features

**Figure 7:**
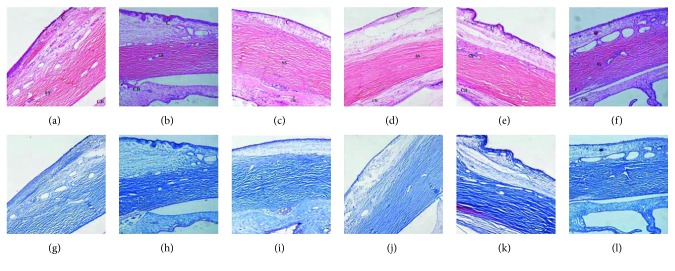
Histological features of filtering positions after 56 days (magnification ratio ×100). (a–f) Hematoxylin and eosin (H&E) staining, (g–l) Masson staining of the tissue sections. In the control (a and g) and 5-fluorouracil (5-Fu) treatment groups (c and i), the subconjunctival fibrous scar tissues were dense. The collagen deposition was relatively loose in the bevacizumab (BVZ) treatment group (b and h), and the structures of filtering blebs could not be distinguished in the three groups. In the mitomycin C (MMC) (d and j), 5-FU + BVZ (e and k), and MMC + BVZ (f and l) treatment groups, the subconjunctival collagen deposition was spare, and the residual filtering bleb structure was observed in all these groups. CB, ciliary body; C, conjunctiva; SS, scleral excision position.

H&E and Masson staining of the tissue sections of the 56-day filtering blebs showed histological features and subconjunctival collagen deposition in the control (Figures 7(a) and 7(g)), BVZ (Figures 7(b) and 7(h)), 5-Fu (Figures 7(c) and 7(i)), MMC (Figures 7(d) and 7(j)), 5-FU + BVZ (Figures 7(e) and 7(k)), and MMC + BVZ (Figures 7(f) and 7(l)) treatment groups.

#### 3.2.4. mRNA Quantitation of Type I Collagen Fiber and Fibronectin in Filtering Blebs

**Figure 8:**
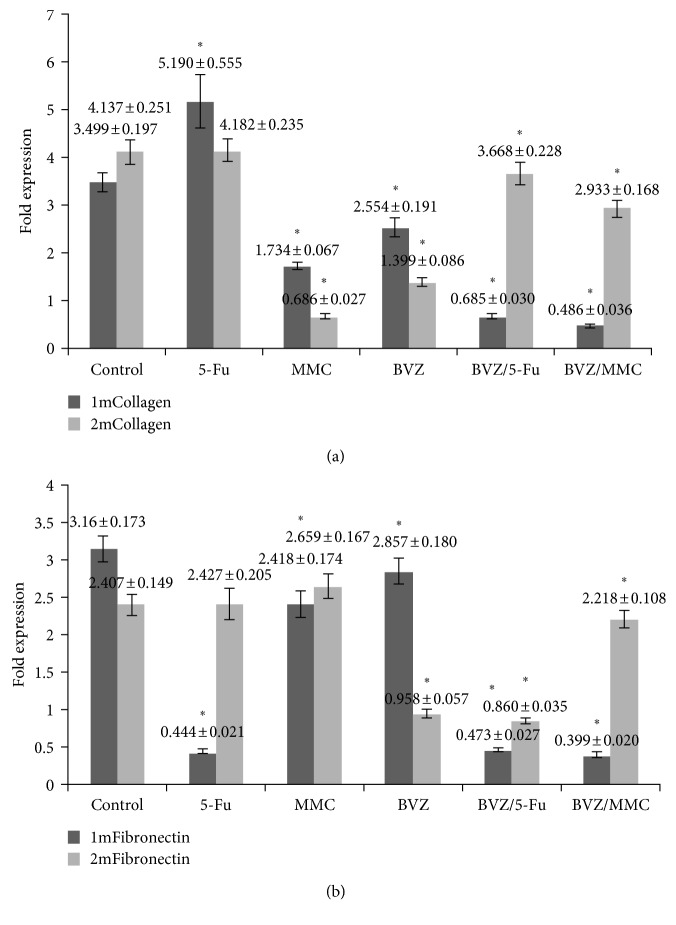
mRNA expression levels of fibrotic markers in the tissues of filtering blebs. In the bevacizumab (BVZ), 5-fluorouracil (5-Fu) + BVZ, and mitomycin C (MMC) + BVZ groups, the mRNA expression levels of type I collagen (a) and fibronectin (b) in filtering blebs were significantly lower than those in the control group. The data in the MMC and 5-Fu treatment groups were not significantly different from those in the control group after both 1 and 2 months (^*∗*^*P* < 0.05).

In the BVZ, 5-Fu + BVZ, and MMC + BVZ groups, the mRNA expression levels of type I collagen fiber (Figures 8(a)) and fibronectin (Figure 8(b)) in the filtering blebs after 1 and 2 months were significantly lower than those in the control group.

## 4. Discussion

This study observed the safety and the antiscarring effects of the use of BVZ + 5-Fu and BVZ + MMC in *in vitro* experiments and experimental GFS. The results showed that the use of BVZ + 5-Fu had better antiscarring effects and provided better cell safety. However, BVZ + MMC showed no significant advantage over MMC alone. The findings provided more reference for the clinical use of adjuncts to inhibit wound healing of the bleb after GFS, contributing to the overall knowledge on wound modulation by the combined use of BVZ and antimetabolic drugs.

The purpose of the GFS surgery was to relieve elevated intraocular pressure by creating an incision to bypass the trabecular meshwork and drain the aqueous humor outward through subconjunctival filtering blebs [37]. The neovascularization of the conjunctiva and migration of the fibroblasts resulted in the proliferation of fibroblasts accompanied by collagen deposits, which directly caused the failure of filtering bleb drainage [38]. Angiogenesis is a process of growing new blood vessels from existing blood vessels. This important process occurs naturally during growth, reproduction, and wound healing to supply nutrients and oxygen to the tissues. VEGF is the most common stimulator for endothelial growth and vascular permeability [39, 40]. It not only regulates fibrosis through angiogenesis but also acts as a mediator in signaling pathways that promote fibroblast migration, proliferation, and collagen production [41, 42].

Seet et al. [43] used mouse models to analyze the time and space of the reaction stages during wound healing after GFS. They found that the tissue reaction after surgery could be divided into two stages: early “acute inflammation” and late “fibrosis” stages. The early acute inflammation stage is characterized by significantly elevated transcriptional expressions of VEGF, chemokines (C-X-C motif), ligand (CXCL), and matrix metalloproteinase (MMP), besides increased infiltration by inflammatory cells. The late fibrosis stage is characterized by the significantly elevated expression of transforming growth factor (TGF)-*β*2 and extracellular matrix genes, whereas the infiltration of inflammatory cells reduced. VEGF-A is the only VEGF subtype significantly elevated during the late stage of wound healing [43], suggesting that it might be involved in the transition of the early to late stage [44]. VEGF signaling is involved in both angiogenesis and fibrosis, two critical processes in scar formation [43, 45]. This has led to studies investigating the ability of anti-VEGF therapy to improve the outcomes of GFS. However, the treatment for one target might not offer adequate benefit for GFS because of the complex wound-healing process. Therefore, this study was concerned not only with the use of BVZ in GFS but also whether the use of BVZ + 5-Fu or BVZ + MMC could better inhibit wound scar formation after GFS. Also, the safety and possible mechanism of action of the aforementioned drugs were investigated.

The present study tested the cytotoxicities of BVZ + 5Fu and BVZ + MMC *in vitro*. Previous studies have used the MTT assay to observe the cytotoxicity of BVZ to ARPE19 cell lines [26]. The cytotoxicity of selected herbal chemicals with potent antiangiogenic therapeutic properties was studied by performing the MTT cell viability/proliferation assay on ARPE19 cells [27]. Therefore, the same method was used in the present study to observe the cytotoxicity of BVZ combined with antimetabolites. The study found that the toxicity of treatment with 0.5 and 5 mg/mL 5-Fu + 2.5 mg/mL BVZ was lower than that of 5-Fu alone. However, the treatment with MMC + 2.5 mg/mL BVZ had toxicity equivalent to that of MMC alone.

Since RPE and endothelial cells are known to express VEGF [46, 47], the impact of combination drugs on RPE cells at the level of VEGF was tested to observe the comprehensive effect of these combinations on wound healing. Further, the study attempted to explore the mechanism of action of combined drugs and examined the cell cycle and apoptosis of RPE cells. The treatment with BVZ + 5-Fu was found to have a significant inhibitory effect on VEGF levels in RPE cells, but BVZ + MMC had no such effect. Both BVZ + 5-Fu and BVZ + MMC blocked the proliferation of RPE cells in the G1/G0 phase and significantly induced RPE cell apoptosis.

Tenon's fibroblast cells are the important mediators in the formation of filtering blebs scar after GFS [48]. *In vitro* studies have reported the inhibitory effect of BVZ on the proliferation and migration of Tenon's fibroblast cells [18, 49]. VEGF signaling is involved in both angiogenesis and fibrosis, two critical processes in scar formation [43, 45]. The present study paid attention to the effect of combined drugs on vascular endothelial cells, which were involved in angiogenesis [50]. The scratch-wound assay is a classic method for studying the spread, proliferation, and migration of vascular endothelial cells, which are typical events in the wound-healing process [27]. Therefore, scratch-wound assay and HUVECs were chosen to observe the effects of BVZ combined with antimetabolites on the migration of vascular endothelial cells. Both BVZ + 5-Fu and BVZ + MMC inhibited the migration of HUVECs.

Animal experiments and small clinical trials showed that BVZ treatment delayed the healing process of filtering blebs after GFS [18, 23, 51]. How et al. [24] performed the subconjunctival injection of BVZ + 5-Fu in an experimental rabbit eye GFS model and observed that the antiproliferative effect of the combined treatment was better than that of each drug alone. In clinical trials, Suh and Kee [52] and Chua et al. [53] also administered BVZ + 5-Fu in GFS. Compared with 5-Fu alone, no significant difference was found in vision, postoperative intraocular pressure, or antiglaucoma drug use. Kahook et al. [54] randomly divided patients with primary open-angle glaucoma into an MMC group and a group treated with MMC and an intravitreal injection of ranibizumab (RBZ). They found that the bleb morphology, such as filtering bleb dispersion and neovascularization, in the combined treatment group was better than that in the single treatment group; however, the intraocular pressure was not different between the groups.

The results of this study showed that BVZ + 5-Fu had a better antiscarring effect, whereas the effect of a single drug was similar to that in the control group. The use of BVZ in combination with antimetabolic drug 5-Fu reduced the VEGF level significantly, which not only inhibited both early and late stages of scar formation but also enhanced the potential anti-inflammatory effects. Therefore, BVZ and 5-Fu might work synergistically [24] and the antiscarring effect was greater than that of the use of the single drug. However, it should be clearly understood that experimental study or clinical outcomes in animal studies may not predict outcomes in human clinical use (such as the CAT-152 trials [55]).

The shortcoming of this study was the lack of the measurement of intraocular pressure. However, the reduction of subconjunctival scars should have a positive effect on the function of filtering blebs.

Since Tenon's fibroblast cells are important mediators in the formation of filtering blebs scar after GFS [48], further studies should include observing the cytotoxicity of combined drugs on human Tenon's fibroblasts (HTF) and the effect of combined drugs on the migration of HTF.

In the present study, the mRNA expression levels on the two fibrotic markers in the BVZ + 5-Fu and BVZ + MMC groups were significantly reduced in the first month, but increased by varying degrees in the second month. This finding suggested that a subsequent study could repeat the BVZ injection in the second month to verify whether it could persistently inhibit the formation of type 1 collagen fibers and fibrin and prolong the survival of filtering blebs [45].

The general and local effects of the subconjunctival injection of BVZ should be further considered. A pharmacokinetic study showed that both subconjunctival and intravitreal injections of BVZ could achieve effective intraocular concentration [21]. Wang and Harasymowycz [56] reported that 3 of 28 patients who received a subconjunctival injection of BVZ during GFS had retinal vein branch occlusion. The combined results of clinical trials showed that patients receiving high doses of RBZ (e.g., 0.5 mg) had a higher incidence of stroke compared with patients receiving a low dose (e.g., 0.3 mg) of intravitreal injection [57]. The safety of anti-VEGF antibodies, including the potential side effects on the eye and entire body, must be more clearly defined.

## 5. Conclusion

The experimental results showed that a single subconjunctival injection of BVZ combined with 5-Fu in experimental GFS has a better antiscarring effect compared with a single drug. BVZ + 5-Fu could significantly prolong the survival time of the filtering bleb with lower cytotoxicity. However, no significant difference was observed between the MMC + BVZ and single MMC groups. The present study provided a more comprehensive reference for clinical improvement in GFS prognosis. The subsequent studies should focus on establishing the mode and frequency of administration of BVZ + 5-Fu and also on further investigating the mechanism of action of BVZ + 5-Fu.

## Figures and Tables

**Table 1:** Primers used in real-time polymerase chain reaction.

Gene name	Primer sequences
Collagen I	Forward: 5′-CAGCCGCTTCACCTACAGC-3′
	Reverse: 5′-TTTTGTATTCAATCACTGTCTTGCC-3′

Fibronectin	Forward: 5′-ACC AAC CTT AAT CCG GGC AC-3′
	Reverse: 5′-TCA GAA ACT GTG GCT TGC TGG-3′

GAPDH	Forward: 5′-AGACAGCCGCATCTTCTTGT-3′
	Reverse: 5′-CTTGCCGTGGGTAGAGTCAT-3′

**Table 2:** Kaplan–Meier analysis and Mantel–Cox log-rank test of the survival time of filtering blebs in each group.

Group	Con	5-Fu	MMC	BVZ	BVZ + 5-Fu	BVZ + MMC
	*P* value	*P* value	*P* value	*P* value	*P* value	*P* value
Con	—	<0.0001	<0.0001	<0.0001	<0.0001	<0.0001
5-Fu	<0.0001	—	<0.0001	0.588	<0.0001	0.002
MMC	<0.0001	<0.0001	—	<0.0001	0.851	0.920
BVZ	<0.0001	0.588	<0.0001	—	<0.0001	0.002
BVZ + 5-Fu	<0.0001	<0.0001	0.851	<0.0001	—	0.794
BVZ + MMC	<0.0001	0.002	0.920	0.002	0.794	—

## Data Availability

All the data supporting the findings of this study are available within the manuscript.
